# Psychometric properties of a sign language version of the Mini International Neuropsychiatric Interview (MINI)

**DOI:** 10.1186/1471-244X-14-148

**Published:** 2014-05-22

**Authors:** Beate Øhre, Hege Saltnes, Stephen von Tetzchner, Erik Falkum

**Affiliations:** 1 Division of Mental Health and Addiction, National Centre for Hearing Impairment and Mental Health, Oslo University Hospital, Oslo, Norway; 2 Department of Psychology, University of Oslo, Oslo, Norway; 3 Department of Research and Development, Division of Mental Health and Addiction, Oslo University Hospital, Oslo, Norway; 4 Institute of Clinical Medicine, University of Oslo, Oslo, Norway

**Keywords:** MINI, Mental disorders, Psychiatric diagnoses, Assessment, Deafness, Hearing loss, Sign language, Psychometrics

## Abstract

**Background:**

There is a need for psychiatric assessment instruments that enable reliable diagnoses in persons with hearing loss who have sign language as their primary language. The objective of this study was to assess the validity of the Norwegian Sign Language (NSL) version of the Mini International Neuropsychiatric Interview (MINI).

**Methods:**

The MINI was translated into NSL. Forty-one signing patients consecutively referred to two specialised psychiatric units were assessed with a diagnostic interview by clinical experts and with the MINI. Inter-rater reliability was assessed with Cohen’s kappa and “observed agreement”.

**Results:**

There was 65% agreement between MINI diagnoses and clinical expert diagnoses. Kappa values indicated fair to moderate agreement, and observed agreement was above 76% for all diagnoses. The MINI diagnosed more co-morbid conditions than did the clinical expert interview (mean diagnoses: 1.9 versus 1.2). Kappa values indicated moderate to substantial agreement, and “observed agreement” was above 88%.

**Conclusion:**

The NSL version performs similarly to other MINI versions and demonstrates adequate reliability and validity as a diagnostic instrument for assessing mental disorders in persons who have sign language as their primary and preferred language.

## Background

Language and communication are fundamental for diagnosing most mental disorders. Most assessment instruments are designed for use with hearing individuals, and many deaf and severely hard-of-hearing people with psychiatric illness may receive incorrect diagnoses because of challenges in communication between patients and professionals [1-3]. The resulting lack of diagnostic precision may have serious bearing on treatment adequacy and quality. In Norway and other countries, there is a need for valid and reliable instruments for assessment of mental disorders in deaf and severely hard-of-hearing persons who have sign language as their primary and preferred language (signers).

The Mini International Neuropsychiatric Interview (MINI) was developed to meet the need for a brief and reliable structured diagnostic interview in clinical practice as well as in research [4,5]. In clinical practice, the MINI is considered a supplement and not a substitution for regular diagnostic intake interviews. It is a structured evaluation of most of the major psychiatric conditions [4,5], and was therefore selected for translation into Norwegian Sign Language (NSL).

The MINI includes 23 disorders from the tenth revision of the International Classification of Diseases and Related Health Problems (ICD-10) [6] and from the Diagnostic and Statistical Manual of Mental Disorders, Fourth Edition (DSM IV) [7]. It is organised in diagnostic sections with branching tree logic and two to four screening questions with yes or no responses for each disorder. Additional symptom questions are asked only when a screening question is endorsed [4,8]. Excellent inter-rater and test-retest reliabilities have been reported for the English and French versions [9], as well as good to very good convergent validity relative to the Composite International Diagnostic Interview, CIDI [4] and the Structured Clinical Interview for Diagnostic and Statistical Manual, SCID [5]. In test-retest analyses, kappa values have indicated excellent agreement according to Fleiss [10] for six diagnoses, fair to good agreement for six diagnoses and poor agreement for seven diagnoses, whereas observed inter-rater agreement has been 75% or above [5].

When a psychiatric diagnostic instrument is translated into a new language, reassessment of validity and reliability is necessary. Wordings that seem identical in different languages may nonetheless be differently interpreted, especially when a written text is translated to a sign language [11,12]. This is not just a matter of semantic divergences, but also of divergences related to cultural understanding of concepts, words (signs) and sentences. Development of expressions that both convey the question’s core content and appear meaningful to the patient is vital.

The MINI has been translated into 43 languages, and its validity and reliability have been explored not only for the original English and the French versions, but also for the Spanish [13], Italian [14], Japanese [15], Moroccan [16], Portuguese [17] and Norwegian versions [18] (see Table 1). No sign language version has been reported. Deaf and hard-of-hearing people have been excluded from most mental health research, including validation studies of the MINI. Exclusion criteria cited in such research include “language problems” [4,14,15], “patients who were hearing impaired, not fluent in English” [19] and “could not be interviewed due to language barriers” [18].

**Table 1:** Studies on the validity and reliability of the MINI

**Study & language**	**Participants**	**Validation**	**Reliability-analysis**	**Results, depressive and anxiety disorders**	**Concordance; Cohen’s kappa mean & range**
Sheehan et al. [5] English	Psychiatric patients N = 308 Non-patients, N = 62 Total N = 370	SCID-P^1^	Test-retest and inter-rater analyse	Concordance between MINI-CR and SCID-P^1^ diagnoses	Twenty-two diagnostic categories analysed
				Major depressive disorder kappa:0.84, observed agreement: 92%	Two diagnoses occurred in less than 5% of participants
				Inter-rater (kappa) 1.00, Retest (kappa) 0.87 Lifetime panic disorder, kappa: 0.80, observed agreement: 91%	Kappa mean: 0.67, range: 0.43-0.90
				Inter-rater (kappa)0.97, Retest (kappa) 0.79	
Lecrubier et al. [4] French	Psychiatric patients N = 260 Non-patients, N = 50 Total N = 310	CIDI^2^	Test-retest and inter-rater analyses	Concordance between MINI and CIDI^2^ diagnoses	Seventeen diagnostic categories analysed
				Major depressive disorder (kappa) 0.73, observed agreement: 87% Test-retest (kappa) 0.83	Four diagnoses occurred in less than 5% of participants
				Lifetime panic disorder (kappa) 0.68, observed agreement: 92% Test-retest (kappa) 0.76.	Kappa mean: 0.66, range: 0.36-0.82
				Inter rater, all diagnoses (kappa) 0.88-1.00	
Bobes [13] Spanish	Primary health care patients N = 126	Expert opinion	Inter-rater analysis	Major depression, sensitivity 0.94, specificity 0.62	No Cohen’s kappa values available
				Generalised anxiety disorder, sensitivity 0.92, specificity 0.65	
Pinninti et al. [19] English	Psychiatric outpatients N = 111	Expert opinion		Diagnostic agreement in 58% of the cases In 33% the disagreement was of substantial nature MINI diagnosed more co-morbid conditions	No Cohen’s kappa values available
Rossi et al. [14] Italian	Psychiatric outpatients N = 50		Inter-rater and test-retest analyses	Major depressive disorder, current: Inter-rater reliability (kappa) 0.96. Test-retest (kappa) 0.46	Thirty-one diagnoses analysed Inter-rater kappa mean: 0.85, range: 0–1.00
				Major depressive disorder, recurrent: Inter-rater reliability (kappa) 0.84. Test-retest (kappa) 0.36	Test-retest kappa mean: 0.41, range: 0–1.00
				Panic disorder lifetime: Inter-rater reliability (kappa) 0.88. Test-retest (kappa) 0.49	Information on diagnoses occurring in less than 5% of participants not available
Kadri et al. [16] Moroccan	Psychiatric patients N = 175 Non-patients, N = 50 Total N = 225	Expert opinion	Inter-rater and test-retest analyses	Concordance between MINI and expert diagnoses Major depressive disorder (kappa) 0.95, observed agreement: 99%	Thirteen diagnostic categories analysed Four diagnoses occurred in less than 5% of participants
				Social phobia (5% of participants) (kappa) 0.91, obs. agreement: 94%	Kappa mean: 0.91, range: 0.79-0.95
				Inter-rater and test-retest reliability (kappa) all above 0.80	
Otsubo et al. [15] Japanese	Psychiatric inpatients ^A^N = 82 ^B^N = 169	^A^SCID-P^1 B^ Expert opinion	Inter-rater and test-retest analyses	Concordance between MINI and ^A^ SCID-P^1/B^ expert opinion	^A^ Thirteen diagnostic categories analysed Four diagnoses occurred in less than 5% of participants
				Major depressive disorder,	Kappa mean: 0.71, range: 0.49-0.93
				^A^ kappa: 0.85, obs. agreement: 93%	^B^ Eleven diagnostic categories analysed Seven diagnoses occurred in less than 5% of participants
				^B^ kappa: 0.36, obs. agreement: 69%	Kappa mean: 0.34, range: 0.03-0.69
				Panic disorder, ^A^ kappa: 092, obs. agreement: 98%	
				^B^ kappa: 0.53, obs. agreement: 89%	
				MINI test-retest, kappa: 0.80, obs. agreement: 91%	
				MINI Inter-rater, kappa: 0.94, obs. agreement: 97%	
Marques & Zuardi [17] Portuguese	Primary health care patients N = 120	SCID^3^		Concordance between MINI and SCID^3^ diagnoses Depressive disorders, kappa: 0.75, obs. agreement: 92%	Eight diagnostic categories analysed Four diagnoses occurred in less than 5% of participants
				Anxiety disorders, kappa: 0.81, obs. agreement: 94%	Kappa mean: 0.66, range: 0–0.85
Mordal et al. [18] Norwegian	Psychiatric patients, acute ward N = 38		Test - retest analysis	Concordance of MINI diagnoses in test-retest	Twenty diagnostic categories analysed Six diagnoses occurred in less than 5% of participants
				Major depressive episode, kappa: 0.82, obs. agreement: 92%	Kappa mean: 0.51, range: 0–1.00
				Panic disorder, lifetime, kappa: 0.84, obs. agreement: 92%	

^1^The Structured Clinical Interview for Diagnostic and Statistical Manual III-R Patient version.

^2^The Composite International Diagnostic Interview.

^3^The Structured Clinical Interview for Diagnostic and Statistical Manual IV.

Sign languages are natural languages that have evolved through use by deaf and hard-of-hearing people. Utterances are produced with the hands and face and are visually received and decoded [20,21]. Sign languages share many linguistic characteristics with spoken languages, but also have characteristics that are specific to the manual-visual modality [20,21]. Most deaf adults use written language and consider it their second language; however, a considerable percentage of congenitally deaf people struggle with written texts [22,23].

To reliably diagnose mental illness in signing persons, clinicians must have sufficient sign language competence and thorough knowledge of deaf culture. They must also have a deep understanding of the potential psychosocial consequences of profound hearing loss, the accompanying language and communication challenges, and the obstacles encountered by many deaf and hard-of-hearing people in society. If these requirements are not met, clinicians may misinterpret the patient’s utterances as symptoms of mental illness or thought disturbances, or may overlook symptoms of mental illness because they are not communicated in the expected manner.

The present study investigates the functionality of a NSL version of the MINI. The main research question is whether this version functions in the same manner with signing deaf and hard-of-hearing patients as do other versions of the MINI with hearing patients.

## Methods

The present investigation is part of a comprehensive study of mental health in individuals referred to specialised psychiatric units for deaf and severely hard-of-hearing patients, and includes patients using different modes of communication and language.

### Participants

Deaf and severely hard-of-hearing signers who were referred to the unit for adults at the National Centre for Mental Health and Hearing Impairment, Oslo University Hospital and the Regional Centre for Mental Health and Hearing Impairment, St. Olav’s Hospital, Trondheim were asked to participate in the study. Exclusion criteria were age below 18 years, spoken language as the main form of communication, dual sensory loss requiring tactile communication, acute and severe psychiatric or somatic illness, and referral for reasons other than assessment of mental disorder. Information was given in NSL or other forms of visual communication preferred by the person, and written consent was obtained from those who decided to participate.

Eighty-eight deaf and severely hard-of-hearing adults were referred to the two specialised mental health services in 2010 (total count of referrals throughout Norway was 120 persons). Twenty-eight of the 88 patients did not meet the inclusion criteria and five did not appear for their appointments. The remaining 55 patients were asked to participate in the study; 10 did not consent and four did not complete all the assessments. The sample therefore comprised 41 patients, or 75% of those who fulfilled the inclusion criteria. Table 2 shows background information for the participants. There were twice as many female as male participants (mean age, 36 years ± 14.1). Most participants had less than a college education, and one-third held a job or was studying.

**Table 2:** Participant information (N = 41)

Number of females	29 (71%)
Age in years	mean: 36, SD 14.1, median: 34, range: 18–83
Decibel loss in the better ear	mean: 84.9, SD 13.8, median: 90, range: 56–110
Marital status	
Single	22 (53.7%)
Married and cohabiting	15 (36.5%)
Divorced, separated & widowed	4 (9.8%)
Ethnicity	
European/Caucasian	37 (90.2%)
Asian	4 (9.8%)
Education	
High school and equivalent	30 (73.2%)
College degree and above	11 (26.8%)
Source of income	
Paid work and study loans	13 (31.7%)
Social welfare and pension	28 (68.3%)
^1^GAF F at admission	mean: 55.2, SD 12.4, median: 52, range: 31–76
^2^GAF S at admission	mean: 55.5, SD 11.0, median: 56, range: 21–73

^1^Global Assessment of Function, Function score.

^2^Global Assessment of Function, Symptom score.

### Assessments

The assessments applied in this study were identical to the ordinary diagnostic assessments at intake. The results were documented in case notes from which personal identifiers had been removed. The diagnoses in these case notes were used to validate the diagnoses assessed by the NSL version of the MINI.

### The MINI

The MINI 5.0.0 was translated from Norwegian into NSL in 2008–2009 through an agreement with Dr. Ulrik Malt, who was leading the team that translated the MINI from English to Norwegian [24]. The translation was performed in accordance with internationally acknowledged translation procedures [25,26] as well as specific procedures suggested for translating written and spoken material into sign language [11,22,27]. A bilingual team of hearing and deaf clinicians and researchers translated each item into sign language and sent video-recordings to deaf and hearing bilingual professionals not familiar with the original text, who back-translated the items into written Norwegian. The research team compared the back-translations to the original written text, and if a back-translated item did not correspond to the content and intent of the original item, the item was discussed and rephrased. Consensus regarding the original text and back-translations was obtained before any item was included in the final video-recorded version. The video-recorded version was subsequently used to instruct and train the experienced MINI assessors, who were signers, to pose the questions of the MINI through uniform Norwegian Sign Language expressions.

### Clinical interview (Expert opinion)

The standard intake interview used at Oslo University Hospital, Clinic of Mental Health and Addiction, was used as expert opinion. It was extended to include questions about background factors and life events that may affect the participant’s vulnerability to a mental disorder. These questions include cause of hearing loss, primary language and communication mode, communication environment in childhood, educational setting and experiences related to growing up with hearing loss.

### Procedure

The clinical interview was conducted by clinical psychologists and psychiatrists, who had long experience with deaf and severely hard-of-hearing patients, were skilled in sign language, and had extensive knowledge about deafness and deaf culture. The communication with the participants was in NSL and was adapted to match the communication style of the participant as needed. The MINI interview was conducted in NSL by experienced MINI assessors.

Each participant was first seen by the team that would conduct the clinical intake interview (expert opinion). The MINI assessment was completed by another team consisting of a deaf therapist who is a native signer who posed the questions to the patients, and either a psychiatrist or a specialised clinical psychologist who guided the deaf co-therapist and the participant through the MINI interview and made the diagnostic decisions. Sometimes, the latter professional would also ask questions to obtain additional information. The two diagnostic teams received the referral information but did not share other information, and were blind to each other’s diagnoses. The two assessments were scheduled with minimal time lag between them. All assessment sessions were video-recorded, except those for three participants who did not want to be recorded.

To ensure that the clinical intake interviews were in fact appropriate to serve as expert opinion for validating the MINI, inter-rater reliability was computed. Eleven (27%) of the recorded clinical interviews were reassessed by a clinical psychologist who was familiar with the patient population and skilled in NSL. He received the referral information and made assessments based on the videotaped interviews, but was blind to the diagnoses given in the assessment. The same procedures were used to compute inter-rater reliability for eight (20%) of the MINI interviews. The patients were drawn randomly, but because the psychologist had to be blind to the person’s diagnoses, the same patient could not be reassessed twice. To prevent resampling, the patients whose intake interviews had been used for reassessment were excluded before the random subsample was drawn.

### Ethics

Study inclusion required informed consent. Participants were informed that they could withdraw from the study at any time and have their videos erased, without any consequences for their treatment. Any participant who might experience additional burden as a result of participation in the study would be offered appropriate support and counselling. The study was approved by the Regional Committee for Medical and Health Research Ethics (REK) and the Norwegian Data Protection Authority (NDPA).

### Statistical analysis

SPSS version 20 was used to analyse the data. Cohen’s kappa was calculated to assess inter-rater reliability and to estimate the validity of the diagnoses assessed by the MINI. According to Shrout [28], kappa values above 0.80 indicate substantial agreement, 0.61–0.80 moderate agreement, 0.41–0.60 fair agreement, 0.11–0.40 slight agreement, and below 0.10 virtually no agreement. Approximate 95% confidence intervals were generated by multiplying the standard error of kappa by 1.96. The observed agreement was the frequency of two raters’ agreement on whether a disorder was present [29].

## Results

The mean length of the MINI interview was 58 min (range 32–88). The most prevalent diagnoses assessed by expert opinion were major depressive disorder (n of diagnoses = 22; 54%) and anxiety disorder (n of diagnoses = 12; 29%). No participant met the criteria for mania, agoraphobia, obsessive compulsive disorder, anorexia nervosa or bulimia nervosa.

Twenty-eight participants (68%) received a diagnosis by both expert opinion and the MINI, with an average of 1.2 diagnoses according to expert opinion and 1.9 according to the MINI. Thirteen participants (32%) did not meet the criteria for any diagnosis on the MINI. Two of them did not receive a diagnosis by expert opinion—four were considered to have major depressive disorder—and seven were diagnosed with disorders of psychological development, mild mental retardation, personality disorder or disorder of severe stress and adjustment, which are included in ICD 10, but not in the MINI.

There was agreement about 49 (64%) of the 77 diagnoses given to the 41 participants.

In five conditions (6%) MINI gave no diagnosis and expert opinion assessed major depressive disorder, and in 13 conditions (17%) with a MINI diagnosis expert opinion assessed “no diagnosis”. Nine conditions (12%) were assigned a diagnosis included in the MINI by the MINI interview and a diagnosis not included in the MINI by the clinical interview. Last, there was major disagreement about one condition (1%), which was diagnosed as drug dependence by the MINI and as major depressive disorder by expert opinion (Additional file 1: Table S1).

Table 3 shows the concordance between the MINI and expert diagnoses. The participants’ conditions covered a wide range of diagnoses, and the two gross diagnostic categories of depressive disorders and anxiety disorders occurred in numbers sufficient to conduct kappa analyses. Four diagnoses were assigned to fewer than 5% of the participants. The point estimate of Cohen’s kappa was 0.46 for depressive disorders, indicating fair agreement according to Shrout [28], and 0.72 for anxiety disorders, indicating moderate agreement. The observed agreement between the raters was 73% and 88%, respectively (Table 4).

**Table 3:** Concordance between the Norwegian sign language version of the MINI and expert opinion, all patients’ diagnoses (N = 41)

	**MINI assessment**			
**Expert opinion**	**- -**	**- +**		
	**+ -**	**+ +**		
**Disorders assessed in the present study (sorted as in MINI)**			**Cohen’s kappa (95% CI)**	**Observed agreement**
Major depressive disorder	17	3	0.51 (0.26-0.77)	76%
	7	14		
Dysthymia*	37	3	0.38 (0.00-0.91)	93%
	0	1		
Panic disorder	31	7	0.39 (0.07-0.72)	83%
	0	3		
Social anxiety disorder	34	3	0.69 (0.37-1.00)	93%
	0	4		
Posttraumatic stress disorder	37	2	0.36 (0.07-0.93)	93%
	1	1		
Alcohol dependence & abuse	38	1	0.79 (0.39-1.00)	98%
	0	2		
Substance abuse*	37	3	0.38 (0.00-0.91)	93%
	0	1		
Psychotic disorders*	40	0	1.00 (1.00)	100%
	0	1		
Generalised anxiety disorder	37	2	0.00 (0.00)	90%
	2	0		
Antisocial personality disorder*	39	1	0.66 (0.03-1.00)	96%
	0	1		

*Diagnoses occurring in less than 5% of the participants according to expert opinion.

**Table 4:** Concordance between the Norwegian sign language version of the MINI and expert opinion, all patients’ diagnoses (N = 41)

	**MINI assessment**			
**Expert opinion**	**- -**	**- +**		
	**+ -**	**+ +**		
**Psychiatric disorders**			**Cohen’s kappa (95% CI)**	**Observed agreement**
Depressive disorders^1^	14	5	0.46 (0.19-0.73)	73%
	6	16		
Anxiety disorders^2^	26	5	0.72 (0.4-0.94)	88%
	0	10		

^1^Depressive disorders include major depressive disorder, current; major depressive disorder, recurrent; and dysthymia.

^2^Anxiety disorders include panic disorder, social anxiety disorder, post-traumatic stress disorder, and generalized anxiety disorder.

Twenty-six per cent of the participants underwent the two diagnostic interviews during the same day. The median number of days between the two interviews was 19 (range 0–115). The point estimate of Cohen’s kappa for depressive disorders was 0.46 for all participants, 0.50 for those who underwent the two interviews within 19 days, and 0.42 for those who waited longer than 19 days. For anxiety disorders the corresponding kappas were 0.72, 0.77, and 0.63.

Eleven participants who were diagnosed with a total of 17 conditions were used in the inter-rater reliability analysis of the diagnoses assessed by expert opinion; there was agreement about 11 conditions (65%). There was disagreement about six conditions (35%) concerning co-morbidity. For two conditions (12%), the initial expert opinion gave a diagnosis and the second rater did not; in another two conditions (12%), the reverse occurred. There was also major diagnostic disagreement about two additional conditions (12%) of co-morbidity. In some cases of disagreement, the second rater’s expert opinion diagnosis was in agreement with the MINI diagnosis and in conflict with the expert opinion. For instance, in one case, both the second rater and the MINI identified drug use, whereas the first expert diagnosed personality disorder (Additional file 2: Table S2).

Cohen’s kappa was computed for the two gross diagnostic categories “depressive disorders” and “anxiety disorders”. The point estimate of Cohen’s kappa in the analysis of the open clinical interview diagnoses was 0.81 for depressive disorders, indicating substantial agreement; the observed agreement was 91% (Table 5). For anxiety disorders, the point estimate of kappa was 0.44, indicating fair agreement, whereas the observed agreement was 73%.

**Table 5:** Inter-rater reliability estimates of diagnoses assessed by the initial clinical intake interview and by the second rater (N = 11)

	**Second rater**			
**Expert opinion**	**- -**	**- +**		
	**+ -**	**+ +**		
**Psychiatric disorders**			**Cohen’s kappa (95% CI)**	**Observed agreement**
Depressive disorders^1^	4	1	0.81 (0.47–1.00)	91%
	0	6		
Anxiety disorders^2^	5	1	0.44 (0.00-0.97)	73%
	2	3		

^1^Depressive disorders include major depressive disorder, current; major depressive disorder, recurrent; and dysthymia.

^2^Anxiety disorders include panic disorder, social anxiety disorder, post-traumatic stress disorder, and generalized anxiety disorder.

In the inter-rater analysis of the diagnoses assessed by MINI, eight participants were diagnosed with 13 conditions; there was agreement about nine conditions (69%). In two conditions (15%) diagnosed by the initial MINI team as alcohol dependence and major depressive disorder, the second rater found no diagnosis. One condition (8%) was assessed as a dissocial personality disorder by the initial MINI team and as a psychotic disorder by the second rater, and one condition (8%) was diagnosed as major depressive disorder by the initial MINI team and as a dissocial personality disorder by the second rater (Additional file 3: Table S3).

For the MINI assessments, the point estimate of Cohen’s kappa was 0.71 for depressive disorders, indicating moderate agreement, and the observed agreement was 88% (Table 6). For anxiety disorders, the agreement was complete (kappa = 1.00, observed agreement = 100%). Four instances of inter-rater disagreement mirrored the disagreement between expert opinion and the MINI diagnoses in the validation analysis.

**Table 6:** Inter-rater reliability estimates for diagnoses assessed by the initial MINI assessment and by the second rater (N = 8)

	**Second rater**			
**MINI assessment**	**- -**	**- +**		
	**+ -**	**+ +**		
**Psychiatric disorders**			**Cohen’s kappa (95% CI)**	**Observed agreement**
Depressive disorders^1^	5	0	0.71 (0.21–1.00)	88%
	1	2		
Anxiety disorders^2^	5	0	1.00 (1.00)	100%
	0	3		

^1^Depressive disorders include major depressive disorder, current; major depressive disorder, recurrent; and dysthymia.

^2^Anxiety disorders include panic disorder, social anxiety disorder, post-traumatic stress disorder, and generalized anxiety disorder.

## Discussion

The MINI and expert diagnoses in deaf and severely hard-of-hearing signers referred for psychiatric evaluation show fair to substantial agreement. For validation against expert opinion, the kappa estimates in the present study are higher than those of Otsubo et al. [15] but lower than those of Kadri et al. [16]. Pinninti et al. [19] and Bobes [13] also used expert opinion for validation but did not offer kappa values.

The kappa scores were lower than those of studies that have validated MINI against SCID-P [5,15,17] and CIDI [4]. This is in line with other validation studies [13,15,19]. The kappa values in validations of the MINI against expert opinion were generally lower than when the MINI has been validated against a structured diagnostic instrument such as SCID-P or CIDI [4,5,15,17]. Only Kadri et al. [16] report relatively high concordance with expert opinion. The open clinical interview and the structured MINI interview represent different approaches to assessing mental disorders, and complete agreement therefore cannot be expected. Structured psychiatric interviews are designed to elicit information about core symptoms of a set of mental disorders, and the concordance of two structured interviews is therefore likely to be higher than for a structured and an open interview. The diagnostic reflections of experts using an open diagnostic interview are likely more diverse than the focused questions and selected list of disorders in the structured interviews.

The concordance of diagnoses was examined by two different methods because the sample size limited the use of kappa statistics. A broad range of diagnoses was found, some with very low frequency. Cohen’s kappa is a conservative indicator and functions best with larger samples. It is unlikely to be reliable when a diagnosis is assessed in fewer than five per cent of a sample [30]. Only “depressive disorders” and “anxiety disorders” were identified frequently enough to assess reliable kappa scores. Matrixes were therefore drawn to show the distribution of diagnoses assessed with the two diagnostic methods and in the inter-rater reliability procedure. This concordance analysis indicates disagreement in approximately one-third of the diagnoses, with most disagreements related to the participants’ second diagnosis (co-morbidity). This finding applies to the validation of MINI versus expert opinion as well as to the inter-rater reliability of the expert diagnosis.

The main question in the present study is whether the NSL version of the MINI functions in the same manner as do other versions of the MINI when compared with expert opinion. Some of the lack of agreement between the two approaches may be caused by differences in diagnostic options. The MINI includes a limited number of diagnoses, and assessors will have to conform to those diagnoses or assign “no diagnosis”. Relying on the MINI for diagnostic assessment may therefore influence the direction of the evaluation, as clinicians attempt to comply with the diagnoses at hand. Clinicians who conduct an open interview have a wider range of diagnoses to consider, which again may shape the direction of their clinical line of thought and the resulting diagnosis. The fact that the kappa values in this study were in the lower range compared with other validation studies may also be partly explained by the presence of less severe disorders and fewer symptoms among the outpatients in this study than among the inpatients and acute ward patients assessed in most other studies (see Table 1).

The MINI indicated a larger number of co-morbid conditions than did the expert opinion. Similar results have been reported in other studies [15,19] and suggest that the structure of the MINI may make the clinician more attentive to symptoms related to disorders additional to the main diagnosis, and that this attentional shift reduces diagnostic shadowing. This interpretation speaks in favour of including the MINI in routine assessment of signers, as is practiced for hearing patients. On the other hand, assessing severe and complex mental conditions by diagnostic categories exclusively may also increase the probability that symptoms are overlooked.

The MINI was originally developed to meet the need for a structured assessment instrument that could be administered in minimal time. In the present study, the average MINI assessment required almost three times the estimated “less than 20 minutes” [8]. There may be several reasons for this. The interviews were conducted by two therapists, a method requiring collaboration and coordination, which may account for part of the extra time used. Furthermore, as was pointed out by Black and Glickman [3], many deaf patients with mental disorders do not have full mastery of sign language and may struggle in expressing themselves and in comprehending what is being communicated to them, and this may lead to many repetitions. Finally, the interviewer cannot simultaneously take notes and communicate with the patient, and consequently the time needed for taking notes will add to the total interview time [12]. However, the reason for including the MINI as an assessment instrument for deaf and severely hard-of-hearing persons was to improve consistency, not to reduce assessment time.

The two most frequent diagnostic categories in the present study were “depressive disorders” and “anxiety disorders”. This result is similar to that of studies of psychiatric patient populations in general [8,31,32] as well as studies of mental disorders in signing deaf and hard-of-hearing patients [3,33]. To ensure that the open clinical intake interview would serve as expert opinion for the validation of the NSL version of the MINI, these two categories were used to assess inter-rater reliability. A second rater re-diagnosed the participants from video-recordings of the original assessment interviews, a common procedure in reliability studies [30]. To our knowledge, this approach has not been applied before in research with deaf and severely hard-of-hearing persons. It poses quite a few challenges to the second rater, who is forced to follow “the diagnostic route” of the clinician who conducted the initial assessment. This is no problem if the second rater agrees with the first, but second raters cannot collect additional information or follow alternative diagnostic routes when they disagree with the assumption of the original assessor. We do not know whether this issue influenced the concordance estimates in the present study.

The average time span between the two assessments was longer than reported in other validation studies of the MINI. Twenty-six per cent of the participants went through both interviews the same day, which may seem optimal. However, participation was voluntary and cancellations and postponements of appointments were rather common. Furthermore, the participants’ symptoms may have influenced their daily functioning and ability to keep appointments. The centralised service for this geographically scattered patient population is likely to have bearing on the time between assessments. When participants lived far from the outpatient clinic and the assessments could not be made on the same or consecutive days, the result was sometimes a long interval between assessments. However, kappa values for assessments made with short and long intervals were quite similar.

### Limitations

There are some limitations to this study. First, the relatively small sample size, broad range of diagnoses, and low numbers for some diagnoses limit the reliability of kappa statistics. Another disadvantage of kappa statistics is that scores indicating adequate or inadequate reliability may be considered arbitrary. To be stringent, the rather strict classification scheme suggested by Shrout [28] was used.

## Conclusion

The kappa scores in the present validation of the NSL version of the MINI and the observed agreements with expert opinion do not differ substantially from those of other validation studies. The NSL version appears to be a reliable diagnostic interview for assessing mental disorders in signing persons, provided that it is used by professionals with appropriate sign language skills and knowledge about the patient group. The results of the present study therefore encourage translation of the MINI into other sign languages.

## Competing interests

The authors declare that they have no competing interests.

## Authors’ contributions

BØ, HS, SvT, and ER all participated in the design and implementation of the study. BØ and HS designed and led the translation procedures and conducted the assessment of the participants. BØ conducted the data analyses, and SvT and ER contributed in the interpretation of the data. BØ drafted the manuscript, and HS, SvT, and ER critically revised it for important intellectual content. All authors have approved the submitted version.

## Pre-publication history

The pre-publication history for this paper can be accessed here:

http://www.biomedcentral.com/1471-244X/14/148/prepub

## Supplementary Material

Additional file 1: Table S1 Concordance of diagnoses assessed by expert opinion and by the MINI. Click here for file

Additional file 2: Table S2 Inter-rater agreement on diagnoses assessed by two expert opinions. Click here for file

Additional file 3: Table S3 Inter-rater agreement for diagnoses given in two MINI assessments. Click here for file
