# Supplementary material for: Psychometric properties of a sign language version of the Mini International Neuropsychiatric Interview (MINI)
Source: BMC Psychiatry. 2014 May 22;14:148. doi: 10.1186/1471-244X-14-148 (PMC4060880; doi:10.1186/1471-244X-14-148)
Supplement: Additional file 1: Table S1 — Concordance of diagnoses assessed by expert opinion and by the MINI. [file 1471-244X-14-148-S1.pdf]

Additional file 1: Table S1. Concordance of diagnoses assessed by expert opinion and by the MINI

Diagnoses assessed by the MINI

Diagnoses assessed by expert opinion

Diagnoses	1.	2.	3.	4.	5.	6.	7.	8.	9.	10.	11.	12.	13.
1.Major depressive episode, current	**2**	3	1										1
2.Major depressive episode, recurrent	4	**5**								1			4
3.Dysthymia			**1**										
4.Agoraphobia													
5.Social phobias					**4**								
6.Panic disorder				1		**3**							
7.Generalised anxiety disorder				1		1							
8.Post-traumatic stress disorder							1	**1**					
9.Alcohol dependence and abuse									**2**				
10.Drug dependence and abuse										**1**			
11.Psychotic disorders											**1**		
12.Dissocial personality disorder												**1**	
13.No diagnosis		1		1	3	5	1	1		1			**2**
14.Other	2		2			1		1	1	1		1	14

The table includes only the diagnoses that were applied by the assessors. The vertical axis represents the diagnoses in the assessment by the expert opinion. The horizontal axis represents the diagnoses given in the MINI interview. The numbers in the cells show the frequency of each combination of diagnoses. Underlined numbers indicate full agreement.
