# Supplementary material for: Psychometric properties of a sign language version of the Mini International Neuropsychiatric Interview (MINI)
Source: BMC Psychiatry. 2014 May 22;14:148. doi: 10.1186/1471-244X-14-148 (PMC4060880; doi:10.1186/1471-244X-14-148)
Supplement: Additional file 2: Table S2 — Inter-rater agreement on diagnoses assessed by two expert opinions. [file 1471-244X-14-148-S2.pdf]

Additional file 2: Table S2. Inter-rater agreement on diagnoses assessed by two expert opinions

Diagnoses assessed by second expert opinion

Diagnoses assessed by first expert opinion

Diagnoses	1.	2.	3.	4.	5.	6.	7.	8.	9.	10.	11.	12.	13.	14.
1.Mild depressive episode														
2.Moderate depressive episode			1	1										
3.Severe depressive episode														
4.Major depressive episode, recurrent	2			**1**										
5.Dysthymia	1													
6.Social phobias						**1**								1
7.Panic disorder														
8.General anxiety disorder														1
9.Mixed anxiety and depressive disorder							1							
10.PTSD										**1**				
11.Drug dependence and abuse														
12.Emotionally unstable personality disorder											1	**1**		
13.Tic disorders	1													
14.No diagnosis									1			1		**1**

The table includes only the diagnoses that were applied by the assessors. The vertical and horizontal axes represent the diagnoses given by the first and second raters, respectively. The numbers in the cells show the frequency of each combination of diagnoses. Underlined numbers indicate full agreement.
