# Supplementary material for: Psychometric properties of a sign language version of the Mini International Neuropsychiatric Interview (MINI)
Source: BMC Psychiatry. 2014 May 22;14:148. doi: 10.1186/1471-244X-14-148 (PMC4060880; doi:10.1186/1471-244X-14-148)
Supplement: Additional file 3: Table S3 — Inter-rater agreement for diagnoses given in two MINI assessments. [file 1471-244X-14-148-S3.pdf]

Additional file 3: Table S3. Inter-rater agreement for diagnoses given in two MINI assessments

Diagnoses assessed by the second MINI rater

Diagnoses assessed by the first MINI rater

Diagnoses	1.	2.	3.	4.	5.	6.	7.	8.	9.
1.Major depressive episode, current									1
2.Major depressive episode, recurrent	2							1	
3.Social phobia			**2**						
4.Panic disorder				**1**					
5.Alcohol dependence and abuse									1
6.Drug dependence and abuse						**1**			
7.Psychotic disorders									
8.Dissocial personality disorder							1	**1**	
9.No diagnosis									**2**

The table includes only the diagnoses that were applied by the assessors. The vertical axis represents the diagnoses in the initial MINI assessment. The horizontal axis represents the diagnoses given by the second MINI rater. The numbers in the cells show the frequency of each combination of diagnoses. Underlined numbers indicate full agreement.
